# Supplementary material for: Hyperfine-phonon spin relaxation in a single-electron GaAs quantum dot
Source: Nat Commun. 2018 Aug 27;9:3454. doi: 10.1038/s41467-018-05879-x (PMC6110844; doi:10.1038/s41467-018-05879-x)
Supplement: Supplementary file 1 — Supplementary Information [file 41467_2018_5879_MOESM1_ESM.pdf]

Hyperfine-phonon spin relaxation in a single-electron GaAs quantum dot, Camenzind *et al.*

SUPPLEMENTARY MATERIAL
Hyperfine-phonon spin relaxation in a single-electron GaAs quantum dot

Leon C. Camenzind,[1,*] Liuqi Yu,[1,*] Peter Stano,[2,3,4] Jeramy D. Zimmerman,[5] Arthur C. Gossard,[5] Daniel Loss,[1,2] and Dominik M. Zumbühl[1]

[1]*Department of Physics, University of Basel, Basel 4056, Switzerland*

[2]*Center for Emergent Matter Science, RIKEN, Saitama 351-0198, Japan*

[3]*Department of Applied Physics, School of Engineering, University of Tokyo, 7-3-1 Hongo, Bunkyo-ku, Tokyo 113-8656, Japan*

[4]*Institute of Physics, Slovak Academy of Sciences, 845 11 Bratislava, Slovakia*

[5]*Materials Department, University of California, Santa Barbara 93106, USA*

CONTENTS

Supplementary Note 1. In-plane magnetic field alignment 2

Supplementary Note 2. Level positioning algorithm and sensor stabilization 3

Supplementary Note 3. Spin relaxation measurement scheme 7

Supplementary Note 4. Spin-readout fidelity 11

Supplementary Note 5. Definitions and notations for the electron, nuclear spins, and phonons 13

Supplementary Note 6. The spin relaxation rate 16

Supplementary Note 7. Anisotropy of the hyperfine relaxation rate 18

Supplementary Note 8. Dipole matrix elements between the spin opposite states 20

Supplementary Note 9. Numerical implementation 22

Supplementary Note 10. Parameters and fitting of the spin-orbit constants 23

Supplementary References 25

Supplementary Note 1. IN-PLANE MAGNETIC FIELD ALIGNMENT

For the measurements shown, it is crucial to have a good alignment of the external magnetic field **B** with the plane of the 2DEG. Large enough in-plane magnetic field needs to be applied to induce sufficient Zeeman splitting for energy readout. On the other hand, formation of Landau levels due to the perpendicular magnetic fields must be avoided. We extract the out-of-plane angle ξ via Hall measurements using the standard van der Pauw geometry. The Hall coefficient $R_{\mathrm{H},\perp}$ for a perpendicular magnetic field was determined in a separate cool-down. In a parallel field configuration, the finite Hall slope from the out-of-plane field component is $R_{\mathrm{H},\parallel} = R_{\mathrm{H},\perp} \sin(\xi)$ and depends on the tilt of the device. No quantum oscillations were observed up to 10 T, which indicates that the out-of-plane component of the applied magnetic field is very small. We use a piezo-electric rotator (Fig. 1b) to rotate the device in a 4 T magnetic field. In Supplementary Figure 1b, ξ is plotted as a function of ϕ, the angle with respect to [100] (Supplementary Figure 1a). As expected, ξ shows a sinusoidal behavior in ϕ with periodicity of 360°. We find a maximal misalignment of 1.3° close to the crystalline direction [110]. Therefore, we conclude that the effect on our measurements due to the field misalignment is negligible (see Supplementary Note 7).

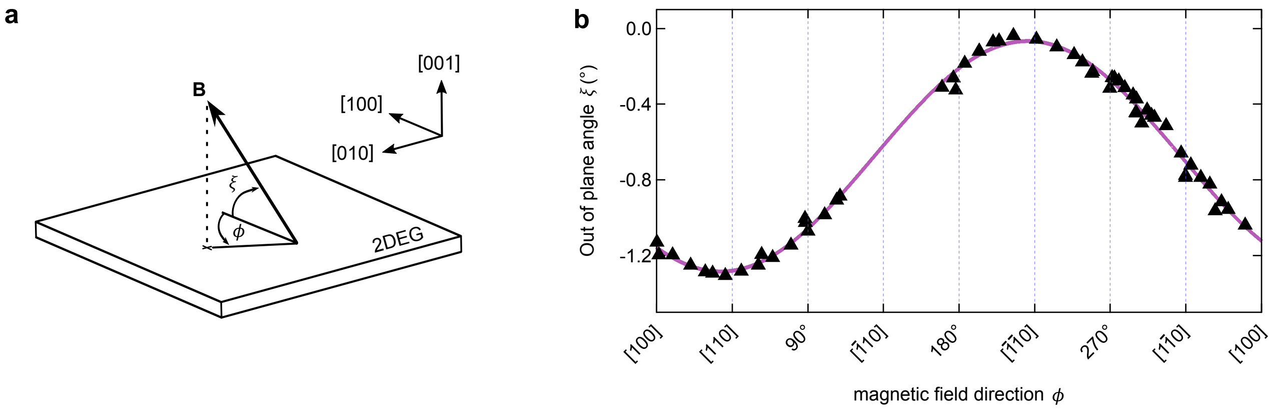

Supplementary Figure 1. In-plane field alignment with 2DEG. a The angles parametrizing the external magnetic field orientation. The small misalignment of the external magnetic field B with the plane of the 2DEG is described by the out of plane angle ξ. The in-plane angle ϕ is defined as the angle with respect to crystal direction [100]. **b** The out-of-plane angle ξ alters as the sample is rotated by the piezoelectric rotator. The data set is very well fitted with a sine of 2π periodicity (purple solid). Between $\phi = 115°$ and 165° the sensor of the piezo-rotator does not encode angles.

Supplementary Note 2. LEVEL POSITIONING ALGORITHM AND SENSOR STABILIZATION

The spin relaxation measurement scheme strongly depends on the associated tunneling rates (see Supplementary Note 3) which themselves strongly depend on the energy detuning of ground-state and the chemical potential μ of the lead[1]. The dot energy levels drift over time, and to compensate for changes in the tunnel rates, we integrate active stabilization protocols. In this section we first give a brief introduction to resonant tunneling before explaining how we exploit this energy dependence for our active level positioning algorithm (LPA)[1]. We then focus on our protocol to maintain the sensitivity of our sensor quantum dot which is also susceptible to fluctuations.

Resonant tunneling of an electron occurs if the occupation probability of the quantum dot is between 0 and 1. In our system, this is observed when the orbital ground-state level of the quantum dot is energetically within the temperature broadening of the 2DEG reservoir (a few $k_{\mathrm{B}}T$) around the lead chemical potential μ. An example is presented in Supplementary Figure 2a where the dot ground state is aligned with μ. Then the occupation probability of the dot is 1/2 and electrons resonantly tunnel from the reservoir to the dot and vice versa. The timescale for this tunneling events is given by the details of the tunnel-barrier and is tunable by the surface gates. Quantitatively the tunneling rate at energy E is $\Gamma(E) = (2\pi/\hbar)T(E)\rho(E)$ with T the transmission coefficient and ρ the density of states in the reservoir[2]. Here, we assume that the tunnel-barrier and the corresponding transmission coefficient T are energy independent for small detuning from μ by a few $k_{\mathrm{B}}T$.

In Supplementary Figure 2b we show an example of resonant tunneling reflected in I_{SQD}, the current through the sensor quantum dot. We use histograms of I_{SQD} (Supplementary Figure 2c) to distinguish the charge states. For a given waiting time t_{w}, we define the total time of the dot being occupied as T_{on}, and being empty as T_{off} respectively. The tunnel rates in and out of the quantum dot are then given by $\Gamma_{\mathrm{in}} = N_{\mathrm{total}}/(2T_{\mathrm{off}})$ and $\Gamma_{\mathrm{off}} = N_{\mathrm{total}}/(2T_{\mathrm{on}})$ with $N_{\mathrm{total}} = N_{\mathrm{on}} + N_{\mathrm{off}}$ the total number of tunneling events during t_{w}. Another method is to histogram the time intervals where the dot is empty (t_{off}) or occupied (t_{on}). These times show an exponential distribution, for example, $\rho_{\mathrm{off}}(t_{\mathrm{off}}) \propto exp(-\Gamma_{\mathrm{in}}t_{\mathrm{off}})$, from where the rates are fitted. In our experiment, the tunnel rates using these methods are in very good agreement. However, the first method avoids errors induced by binning or fitting, thus is preferred for automatized control. In Supplementary Figure 3a-e, the energy diagrams illustrate μ, the ground-state energy of the dot and resonant tunnel rates in (Γ_{in}) and out (Γ_{out}) of the dot for five exemplary situations[3,4]. Here the dot level is controlled by adjusting the voltage on the center plunger gate CP (see Fig. 1a in the main text). In Supplementary Figure 3a, the ground state is well above μ such that an electron on the dot would tunnel out immediately. When the detuning to μ is made smaller, occupied states in the reservoir become resonant with the dot level and elastic tunneling could occur (Supplementary Figure 3b). Because there are more empty than occupied states in the reservoir, the dot is predominantly empty and $\Gamma_{\mathrm{off}} > \Gamma_{\mathrm{on}}$. When the ground state is aligned with μ (Supplementary Figure 3c), $\Gamma_{\mathrm{off}} = \Gamma_{\mathrm{on}}$ and the dot occupation probability is 1/2. Further lowering the dot level reverses the behavior and $\Gamma_{\mathrm{off}} < \Gamma_{\mathrm{on}}$ (Supplementary Figure 3d) until there is no available empty state in the reservoir for the electron to elastically tunnel out of the dot (Supplementary Figure 3e). In our system, we find that inelastic tunneling is strongly suppressed and the electrons are usually trapped for tens of seconds.

Supplementary Figure 3f shows the quantitative dependence of Γ_{in} and Γ_{off} on the detuning from μ. To illustrate that this behavior is explained by the occupation statistics of the lead, the data is fit to a Fermi-Dirac distribution. The knowledge that the rates are distributed accordingly is used for positioning the ground state relative to μ by

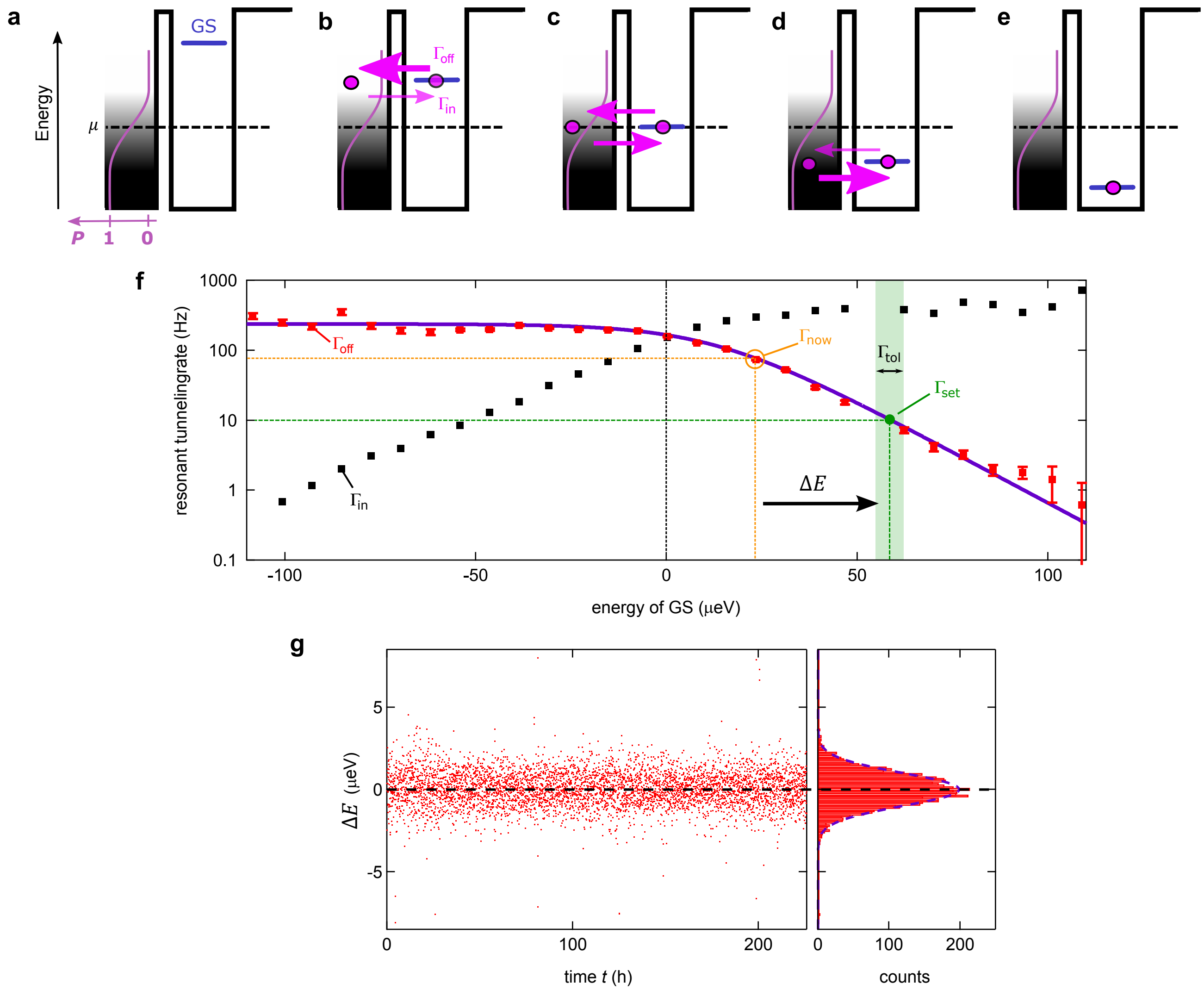

Supplementary Figure 2. Resonant tunneling and discrimination of charge states. a Schematics of resonant tunneling of electrons between the quantum dot and the reservoir. The purple curve indicates the thermal broadening of occupation statistics. This Fermi-Dirac distribution represents the probability of finding an electron in the reservoir as a function of energy. Due to Coulomb blockade we assume no other level is available and the dot is either empty (0) or loaded (1). **b** An exemplary time trace of the resonant tunneling reflected as jumps between two distinguished values of I_{SQD}. As described in the main text, the tunnel rates $\Gamma_{\mathrm{on,off}}$ are calculated by analyzing such resonant tunneling traces. **c** Histogram of the trace shown in **b** exhibits two-level statistics. Due to the large signal-to-noise ratio the charge states (0) and (1) are distinguished with high fidelity. Also, we measure with tunnel rates well below the bandwidth of the charge sensor what minimizes errors due to missed events.

establishing a closed-loop feedback either on the tunnel rates or on the dot occupation probability.

The feedback protocol is illustrated in Supplementary Figure 3f. As shown in the example, the measured tunnel rate off the dot Γ_{now} is feedbacked to adjust the dot level. Therefore, a correction ΔE is calculated and applied to the plunger gate CP (see in Fig. 1a) to restore the set tunnel rate Γ_{set}. This process is repeated until Γ_{now} is within

the tolerance Γ_{tol} around Γ_{set}.

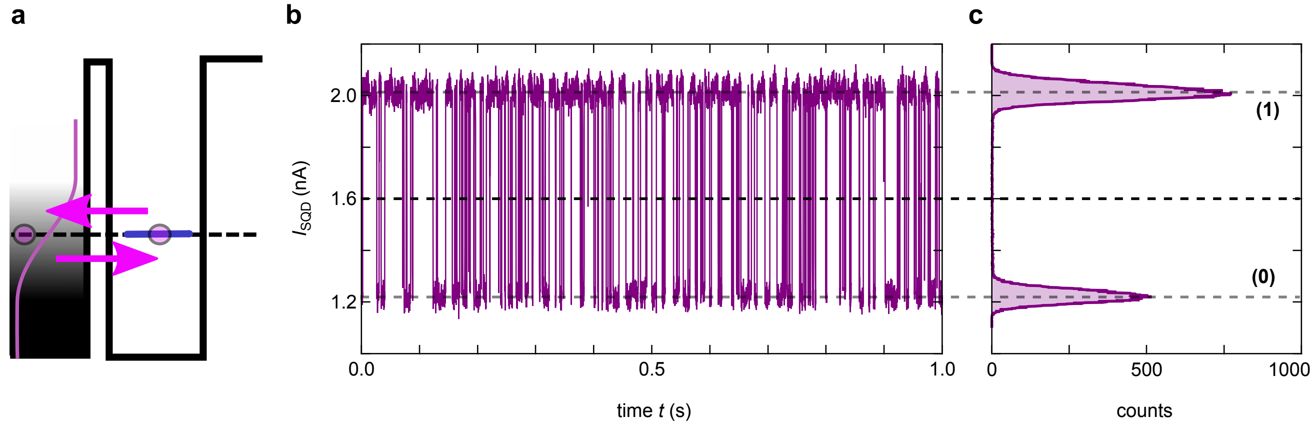

Supplementary Figure 3. Level positioning algorithm (LPA). a-e Schematics of tunnel rates (purple arrows) for different positions of the ground state with respect to μ. The purple curve in the reservoir (left) depicts the probability of finding an electron in the reservoir as a function of energy. **f** Energy dependence of resonant tunnel rate out of the dot Γ_{off} (red square). Γ_{in} exhibits Fermi-Dirac statistics around the regime of resonant tunneling. The electron temperature is around 200 mK. The ground state of the dot drifts over time. Γ_{tol} of Γ_{set} is the tolerance interval, which, in practice, is usually set to be 10% of Γ_{set}, as shown by the green shaded region. For illustration, an example for a correction is shown: if the measured tunnel off rate Γ_{now} is beyond the tolerance interval, the corresponding plunger gate is corrected by ΔE to reset the initial position the ground state. This process is repeated in a closed-loop until Γ_{set} is restored. **g** The stability of the quantum dot is represented by the correction ΔE for one spin relaxation measurement whereas the LPA is performed about every three minutes. A histogram of ΔE is shown on the right side of the panel. The data exhibits a Gaussian distribution centered at 0 with standard deviation of 1.4 μeV. It demonstrates that the dot is very stable.

During the spin relaxation measurements, this feedback is performed about every three minutes. As will be discussed in Supplementary Note 3, it is of great significance to have a small, well known and constant Γ_{off} of the spin ground state to guarantee a reliable spin-to-charge conversion. The dot is usually loaded and in Coulomb blockade when the spin excited state becomes resonant, so that only resonant tunneling with the spin ground state is visible. In reality, due to the thermal broadening, the spin excited state at the smallest fields also contributes to the total resonant tunneling which distorts the measured rates. However, even if the rates are distorted the tunnel off rates of the spin excited state is much larger than for the spin ground state which is needed for the spin-to-charge conversion (see Supplementary Note 3).

In Supplementary Figure 3g, a series of 5000 corrections (ΔE) are shown for a single spin relaxation measurement at 0.7 T over a continuous measuring time of almost 10 days. For this measurement, the spin ground state is maintained at $\Gamma_{\mathrm{off}} = 10\,\mathrm{Hz}$. We record resonant tunneling (Supplementary Figure 2) for 14 s and extract the rates $\Gamma_{\mathrm{in,off}}$. Note that Γ_{off} is equivalent to the background rate Γ_b described in the spin-to-charge conversion in Supplementary Note 3. The LPA allows measurements relying on precise alignment of the dot energy levels for an extended period of time, which is crucial to acquire enough data to provide statistics for extractions of long spin relaxation times.

Next, we turn to corrections of the sensor quantum dot. The best sensitivity is achieved when the sensor is positioned on the steepest point of a Coulomb peak (see Supplementary Figure 4a). To preserve this operation point, a feedback is regularly carried out to compensate for sensor drifts. Before the sensor feedback was carried out, the main dot is slightly detuned from μ to avoid resonant tunneling and a stable I_{SQD} is read. Drift results in changes of the sensor dot energy spectrum indicated in Supplementary Figure 4a. This leads to a change of I_{SQD} and, more importantly, to a reduction in sensitivity ($dI_{\mathrm{SQD}}/dV_{\mathrm{SP}}$). By applying corrections to the sensor plunger SP (Supplementary Figure 4b) in a closed loop, the original sensor operation point is restored. These corrections are calculated with the flank of the Coulomb peak being linearly approximated in V_{SP}. In Supplementary Figure 4b, the evolution of applied voltage on SP, V_{SP}, is shown for the same spin relaxation measurement discussed in Supplementary Figure 3. In contrast to Supplementary Figure 3g, only a few and solely positive corrections were carried out. This unidirectional behavior is often seen but its origin is not clear.

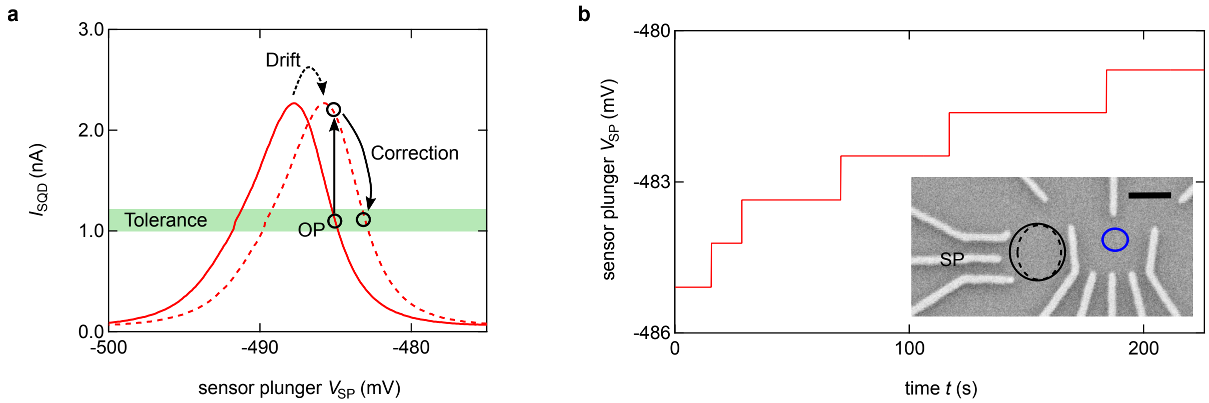

Supplementary Figure 4. Sensor feedback. **a** I_{SQD} of a sensor Coulomb peak (red solid curve) with the sensor operation point (OP, black circle). With time, the Coulomb peak shifts in energy and hence in plunger voltage V_{SP} (red dashed curve). This changes the sensor signal as well as the sensitivity indicated by the vertical arrow. To restore the original operation point, V_{SP} is adjusted until I_{SQD} is once more within a tolerance. **b** The effective voltage on SP for a long measurement showing corrections to compensate drift. The carried-out corrections are of similar magnitude because the feedback is applied as soon as I_{SQD} is out of the tolerance. The inset schematically shows the sensor dot becoming more confined (dashed circle) due to the drift. Scale bar is 200 nm.

Supplementary Note 3. SPIN RELAXATION MEASUREMENT SCHEME

In this section, we present the rate equations describing the three-step pulse measurement scheme used to extract the spin relaxation rate W. This section gives a brief summary of the rate equations solved in Ref. 1.

3a. Ionization

Both spin-up and spin-down states are pulsed well above μ for several ms. If the dot is occupied, the electron will tunnel off so that the dot will be empty or ionized. We choose the duration and the ground state energy detuning such that the ionization probability is more than 99%.

3b. Charge and Relax

In the charge and relax pulse step, both spin states are pulsed below μ (see Supplementary Figure 5a). During the waiting time t_{w}, four pathways are possible: (1) the dot stays empty; (2) an electron tunnels into the spin ground state; (3) an electron tunnels into and stays in the spin excited state; (4) an electron tunnels into the spin excited state and relaxes into the spin ground state. There are other suppressed paths like exchange with the reservoir after loading. Such alternative events are found not to influence the statistics and therefore are neglected. Put simply, the measurement scheme relies on counting electrons taking path (3), which are identified by observing a tunneling out of the spin-excited state during the spin-to-charge conversion.

Under the assumption that the dot is ionized in the beginning of the charge and relax step (see Supplementary Figure 5a), the rate equation for the probability for the dot being empty is

$$\dot{P}_{\mathrm{empty}}(t) = -\Gamma_{\mathrm{in}} P_{\mathrm{empty}} \rightarrow P_{\mathrm{empty}}(t) = e^{-\Gamma_{\mathrm{in}} t}. \qquad \text{(Supplementary Equation 1)}$$

$$\Gamma_{\mathrm{in}} = (\Gamma_e + \Gamma_g) \qquad \text{(Supplementary Equation 2)}$$

Note that $P_{\mathrm{empty}}(t) = 1 - P_L(t)$ with P_L the loading probability during the charge and relax step. Although the individual coupling of the spin excited and ground states to the reservoir, Γ_e and Γ_g, is unknown[5–8], the total coupling Γ_{in} can be obtained by two different methods. Supplementary Figure 5b shows $P_{\mathrm{empty}}(t_{\mathrm{w}})$, the probability distribution of the dot being empty when entering the read-out stage (3c) after waiting time t_{w} in the charge-and-relax stage. This probability is fitted to an exponential function to find Γ_{in}. In the second method, Γ_{in} is obtained by a fit to the histogram of t_{on}'s, the times for an electron to tunnel into the empty dot (Supplementary Figure 5c). The drawback of this method is that in addition to the readout the sensor must also be sensitive during the charge and relax stage to detect t_{on}.We therefore apply a compensation pulse to sensor plunger gate SP (see the inset of Supplementary Figure 4b) to retain sensitivity. This method also allows to obtain Γ_{in} for each waiting time t_{w} individually. As shown in Supplementary Figure 5d, Γ_{in} is independent of t_{w} as expected.

Without considering the contribution due to the thermal excitation from the spin ground state, the rate equation of the probability for an electron being in the spin excited state is $\dot{P}_e = \Gamma_e P_{\mathrm{empty}} - W P_e$. By solving this equation, we find the probability

$$P_e(t) = \frac{\Gamma_e}{\Gamma_{\mathrm{in}}} \cdot \frac{\Gamma_{\mathrm{in}}}{\Gamma_{\mathrm{in}} - W} \cdot (e^{-Wt} - e^{-\Gamma_{\mathrm{in}} t}). \qquad \text{(Supplementary Equation 3)}$$

Note that $\frac{\Gamma_e}{\Gamma_{\mathrm{in}}}$, which is not known, is only a scaling factor.

3c. Read out

When entering the read-out (RO) stage, the probability of the electron still being in the spin excited state after the charge and relax stage is $P_e(t_\mathrm{w})$. In the read-out stage, an electron can leave the spin excited state by either tunneling off the dot with a rate $\Gamma^{\mathrm{RO}}_{\mathrm{off,e}}$ or by relaxing into the spin ground state with spin relaxation rate W. Thus, the rate equation for an electron tunneling out of the spin excited state is $\dot{P_e}^{\mathrm{RO}} = -\Gamma^{\mathrm{RO}}_{\mathrm{off,e}} P^{\mathrm{RO}}_e - W P^{\mathrm{RO}}_e$, which leads to

$$P^{\mathrm{RO}}_e(t) = P_e(t_\mathrm{w}) \cdot e^{-(\Gamma^{\mathrm{RO}}_{\mathrm{off,e}}+W)t}. \qquad \text{(Supplementary Equation 4)}$$

For the spin ground state, either an electron in the spin excited state can relax with rate W or an electron can tunnel into the reservoir with background rate Γ_b. The rate equation is $\dot{P_g}^{\mathrm{RO}} = -\Gamma_b P^{\mathrm{RO}}_g + W P^{\mathrm{RO}}_e$, whereas the spin excited state is involved due to spin relaxation. The solution for this equation is

$$P^{\mathrm{RO}}_g(t) = P_g(t_\mathrm{w}) e^{-\Gamma_b t} + P_e(t_\mathrm{w}) \cdot \frac{W}{\Gamma^{\mathrm{RO}}_{\mathrm{off,e}} + W - \Gamma_b}(e^{-\Gamma_b t} - e^{-(\Gamma^{\mathrm{RO}}_{\mathrm{off,e}}+W)t}), \qquad \text{(Supplementary Equation 5)}$$

where $P_g(t_\mathrm{w}) = 1 - P_e(t_\mathrm{w}) - P_{\mathrm{empty}}(t_\mathrm{w})$ is the probability for an electron to be in the spin ground state when entering the read-out configuration. $P^{\mathrm{RO}}_g(t)$ and $P^{\mathrm{RO}}_e(t)$ are not directly observable in the experiment. But we can detect the timing of tunnel events out of the quantum dot during the readout stage. The probability for an electron tunneling off at time t_{off} in the readout stage is $P^{\mathrm{RO}}_{\mathrm{off}} = \Gamma^{\mathrm{RO}}_{\mathrm{off,e}} P^{\mathrm{RO}}_e(t_{\mathrm{off}}) + \Gamma_b P^{\mathrm{RO}}_g(t_{\mathrm{off}})$ which is equivalent to

$$P^{\mathrm{RO}}_{\mathrm{off}} = \eta \cdot P_\mathrm{e}(t_\mathrm{w}) \left(\Gamma^{\mathrm{RO}}_{\mathrm{off,e}} + W\right) e^{-\left(\Gamma^{\mathrm{RO}}_{\mathrm{off,e}}+W\right)t_{\mathrm{off}}} + \left(P_\mathrm{g}(t_\mathrm{w}) + \frac{W}{\Gamma^{\mathrm{RO}}_{\mathrm{off,e}} + W - \Gamma_b} P_\mathrm{e}(t_\mathrm{w})\right) \Gamma_b e^{-\Gamma_b t_{\mathrm{off}}}$$
(Supplementary Equation 6)

with $\eta = \left(1 - \frac{W\Gamma_b}{\Gamma^{\mathrm{RO}}_{\mathrm{off,e}}\left(\Gamma^{\mathrm{RO}}_{\mathrm{off,e}}+W-\Gamma_b\right)}\right) \frac{\Gamma^{\mathrm{RO}}_{\mathrm{off,e}}}{\Gamma^{\mathrm{RO}}_{\mathrm{off,e}}+W}$, the fraction of electrons in the spin excited state which tunnel out before they relax into the spin ground state. For low fields, $\Gamma^{\mathrm{RO}}_{\mathrm{off,e}} \gg W, \Gamma_b$ such that $\eta \approx 1$ while at high fields $W \sim \Gamma^{\mathrm{RO}}_{\mathrm{off,e}}$ and η is reduced to $\Gamma^{\mathrm{RO}}_{\mathrm{off,e}}/(\Gamma^{\mathrm{RO}}_{\mathrm{off,e}} + W)$. Note that for the measurements presented in Fig. 2 of the main text, $\eta \approx 1$, and it thus has not been involved in the discussion for better readability.

3d. Extraction of W

Supplementary Figure 5e shows exemplary histograms of t_{off} for three waiting times t_w in the charge and relax stage. The counts out of the dot depend on the loading probability $P_L(t_\mathrm{w})$ and the probability to relax into the spin ground state during t_w, W. In first panel of Supplementary Figure 5e, t_w is short compared to $1/\Gamma_{\mathrm{in}} \sim 1kHz$ and the dot is mostly empty when entering the read-out stage. For the next panel, $t_\mathrm{w} > 1/\Gamma_{\mathrm{in}}$ so that P_L is increased. But $t_\mathrm{w} < 1/W$ and there is an increased number of electrons in the spin excited state which have not yet relaxed when entering the read out stage. For the third panel, $P_L \sim 1$ but also $t_\mathrm{w} > 1/W$ such that almost all electrons have relaxed into the spin ground state when entering the read-out stage. The total rate out of the excited state $R = \Gamma^{\mathrm{RO}}_{\mathrm{off,e}} + W$ is independent of t_w, as shown in Supplementary Figure 5f. This allows us to extract ηP_e by fitting (Supplementary Equation 6) to our t_{off} histograms for t_w's. Γ_b is set and fixed by the LPA as mentioned above. For low fields, where $\Gamma^{\mathrm{RO}}_{\mathrm{off,e}} \sim \Gamma_b$, we also explicitly fit Γ_b giving excellent agreement with the values chosen for the LPA.

ηP_e is then plotted as a function of t_w. The spin relaxation rate W can be explicitly found out by fitting $\eta P_\mathrm{e}(t_\mathrm{w})$ to (Supplementary Equation 3). As mentioned above, Γ_{in} is independently obtained from histograms of t_{on} or $P_{\mathrm{empty}}(t_\mathrm{w})$. Note that η is only a scaling factor for (Supplementary Equation 3) and does not affect our ability to extract W. Supplementary Figure 5g shows $\eta P_\mathrm{e}(t_\mathrm{w})$ for selected applied magnetic fields with the respective fits. For the first three panels $W < \Gamma_{\mathrm{in}}$ and the exponential increase in $\eta P_\mathrm{e}(t_\mathrm{w})$ is represented by Γ_{in} while the decay is characterized

by W. For the last panel, $W > \Gamma_{\rm in}$ and the exponential increase is actually given by W and the loading rate $\Gamma_{\rm in}$ is seen in the decay.

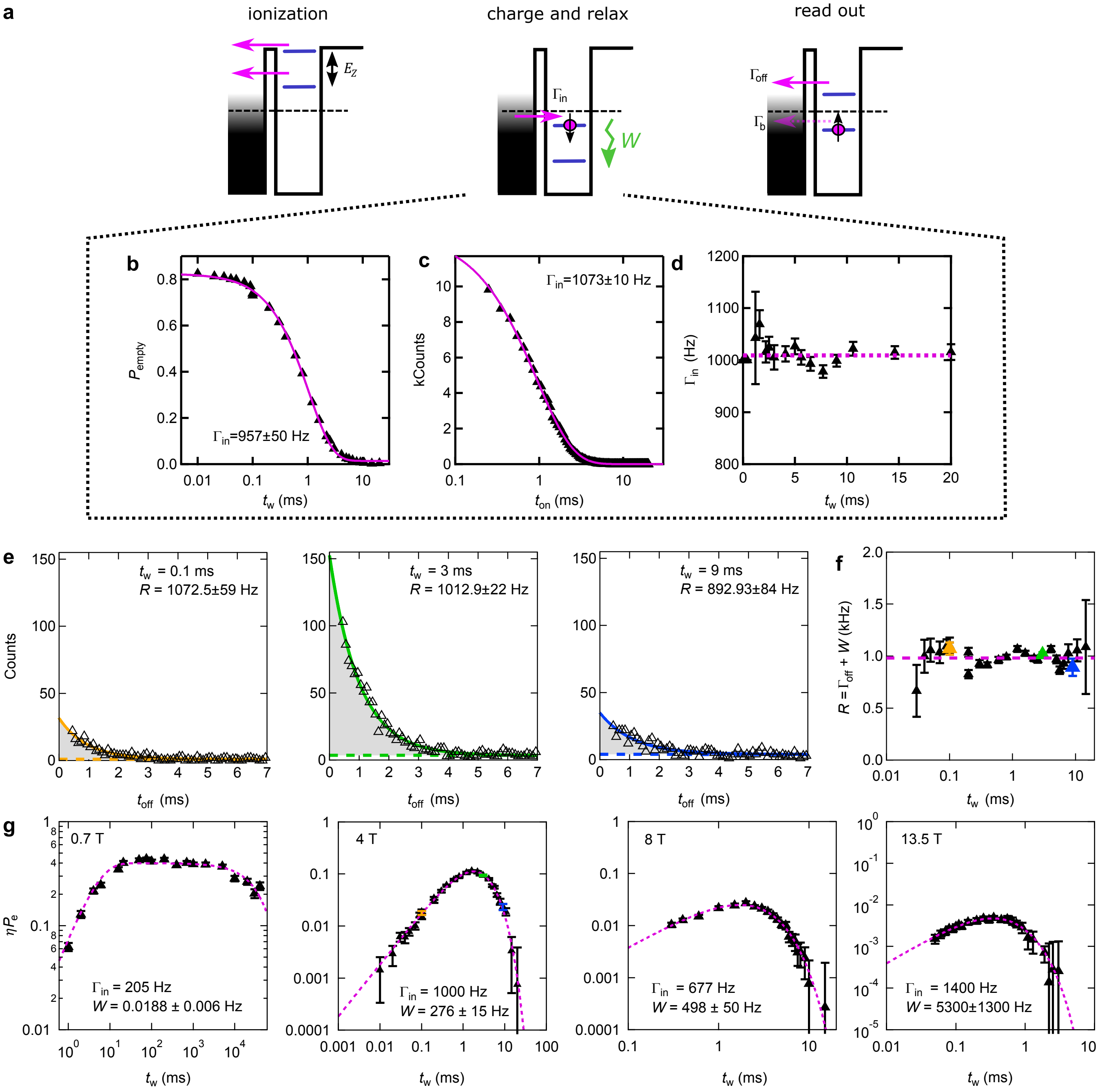

Supplementary Figure 5. T_1 measurement scheme. **a** Schematics of the three-step pulse sequence used for the T_1 measurement for Zeeman energy $E_z = g\mu_B B$ and spin relaxation rate W. Methods to extract $\Gamma_{\rm in}$ by fitting ionization probability $P_{\rm empty}$ for different waiting times $t_{\rm w}$ (**b**) or by fitting the histogram of $t_{\rm on}$ for all data (**c**). **d** shows $\Gamma_{\rm in}$ at various waiting times $t_{\rm w}$ using the second extraction method. It shows that $\Gamma_{\rm in}$ is independent of $t_{\rm w}$. **e** Histograms of tunnel off times $t_{\rm off}$ in the read-out stage for three different waiting times $t_{\rm w}$ exhibit a double exponential distribution with rates $R = \Gamma^{\rm RO}_{\rm off,e} + W$ (solid line) and Γ_b(dashed) as described in the text. **f** R as a function of $t_{\rm w}$. Colored datapoints correspond to colors adopted in **e**. **g** $P_{\rm e}$ for different $t_{\rm w}$ with fits to (Supplementary Equation 3) as described in the text for four different fields. W can be then extracted accordingly. Error bars are fit errors.

Supplementary Note 4. SPIN-READOUT FIDELITY

In this section, we describe a method to calculate the single-shot read-out fidelity. This method is different from the technical read-out fidelity often adopted in systems where detection of tunneling events is difficult[9,10] or re-tunneling into the spin ground state during read-out occurs with speed in the order of the detection bandwidth. For the experiment here, the bandwidth of the sensor exceeds all relevant tunneling rates. Rather, the fidelity here is limited by our capability to distinguish the "blind counts" from electrons either tunneling out of the spin excited state or the spin ground state.

As described in Sec. Supplementary Note 3, we do not assign each tunnel-event in the read-out stage as a spin excited event. Our method takes events from the spin-ground state into account which makes the analysis more generic. Obviously it is beneficial if the tunneling rate out of the spin excited state $\Gamma_e^{\mathrm{RO}} = \Gamma_{\mathrm{off}}$ dominates over the rate out of the spin ground state $\Gamma_g^{\mathrm{RO}} = \Gamma_b$ which is best fulfilled at large Zeeman splittings E_z (see Supplementary Figure 5(e) for an example). At low fields, these rates become closer and the spin read-out fidelity drops.

Using the input from our experimental data, we do excessive simulations of the complete spin relaxation measurement (load and read-out stage) which allows us to track the spin information of a single-shot measurement at any given time (see Fig. Supplementary Figure 6). With these simulations, we generate a complete data set of sensor-current of the pulse-sequences which we run against our analysis algorithms. The fidelity is then based on simulations describing how well the experimental data agree with the rate-equation model (see Sec. Supplementary Note 3). We obtain the spin read-out fidelity by assigning all (simulated) tunnel events counted in the read-out stage before time t^*_{off} as spin excited events and all events afterward as spin-ground state and determine our success rate. The contribution of the ground and excited state during read out are described in Eq. (Supplementary Equation 6) of Sec. Supplementary Note 3 in great detail. We rewrite this formula as

$$P_{\mathrm{off}}^{\mathrm{RO}} = P_{\mathrm{off,e}}^{\mathrm{RO}} + P_{\mathrm{off,g}}^{\mathrm{RO}} = A_e \cdot e^{-(\Gamma_{\mathrm{off}}^{\mathrm{RO}}+W)t_{\mathrm{off}}} + A_g \cdot e^{-(\Gamma_b^{\mathrm{RO}})t_{\mathrm{off}}} \qquad \text{(Supplementary Equation 7)}$$

and describe the fidelity as the ratio of the correctly assigned events to the total counted events

$$F(t^*_{\mathrm{off}}) = \frac{\int_0^{t^*_{\mathrm{off}}} P_{\mathrm{off,e}}^{\mathrm{RO}} dt_{\mathrm{off}} + \int_{t^*_{\mathrm{off}}}^{\infty} P_{\mathrm{off,g}}^{\mathrm{RO}} dt_{\mathrm{off}}}{\int_0^{\infty} (P_{\mathrm{off,e}}^{\mathrm{RO}} + P_{\mathrm{off,g}}^{\mathrm{RO}}) dt_{\mathrm{off}}}. \qquad \text{(Supplementary Equation 8)}$$

Note that $\int_0^{\infty} (P_{\mathrm{off,e}}^{\mathrm{RO}} + P_{\mathrm{off,g}}^{\mathrm{RO}}) dt_{\mathrm{off}}$ does not necessarily equal 1 because of the possibility to enter the read-out stage with an empty quantum dot. Next, we give the explicit expression for the fidelity

$$F(t_{\mathrm{off}}) = \frac{R\left(\frac{e^{-\Gamma_b t_{\mathrm{off}}}(P_{\mathrm{g}}(t_{\mathrm{w}})(-R+\Gamma_b) - P_{\mathrm{e}}(t_{\mathrm{w}})W)}{-R+\Gamma_b} + \frac{\left(1-e^{-Rt_{\mathrm{off}}}\right)P_{\mathrm{e}}(t_{\mathrm{w}})(R\Gamma_{\mathrm{off}} - \Gamma_b(\Gamma_{\mathrm{off}}+W))}{R(R-\Gamma_b)}\right)}{(P_{\mathrm{g}}(t_{\mathrm{w}})R + P_{\mathrm{e}}(t_{\mathrm{w}})(\Gamma_{\mathrm{off}}+W))} \qquad \text{(Supplementary Equation 9)}$$

and the optimal time for the spin state discrimination

$$t^*_{\mathrm{off}} = -\frac{Log\left(\frac{P_{\mathrm{g}}(t_{\mathrm{w}})R\Gamma_b - P_{\mathrm{g}}(t_{\mathrm{w}})\Gamma_b^2 + P_{\mathrm{e}}(t_{\mathrm{w}})\Gamma_b W}{P_{\mathrm{e}}(t_{\mathrm{w}})(RRoff - \Gamma_b Roff - \Gamma_b W)}\right)}{R-\Gamma_b}. \qquad \text{(Supplementary Equation 10)}$$

To prove the validity of our method, we compare the histogram of the total events from the simulations with the histogram obtained from the experiment and find excellent agreement. Fig. Supplementary Figure 6(a) shows an

exemplary data set for an intermediate field of 4 T. Because of decent Zeeman splitting, $\Gamma_{\rm off} \ll \Gamma_b$ and the error of assigning spin ground state events as the spin excited events is small. A maximal fidelity of 99% is found for $t^*_{\rm off} = 3.7$ ms. Note that even for $t^*_{\rm off} = 0$ the fidelity is larger than 50% because as the measurement-scheme enters the read-out stage, the majority of electrons loaded into the spin excited state already relaxed into the spin ground state and will tunnel out with very small rate Γ_b. For lower magnetic fields, the fidelity drops as the ground state contribution significantly affects the read out statistics [see Fig. Supplementary Figure 6(b)]. For the low field measurement presented (0.7 T) we found a maximal fidelity of 81.5%.

Because of spin relaxation, the count of spin excited electrons is always smaller than the spin ground state electrons, which leads to an increased spin readout fidelity. The formalism presented allows to calculate the fidelity with $P_{\rm g}(t_{\rm w}) = P_{\rm e}(t_{\rm w}) = 0.5$. This corresponds the the limit of $\Gamma_{\rm on} \to \infty$ and $t_{\rm w} \to 0$. In this scenario, the fidelity reduces to 98.1% at 4 T and 79% at 0.7 T, respectively.

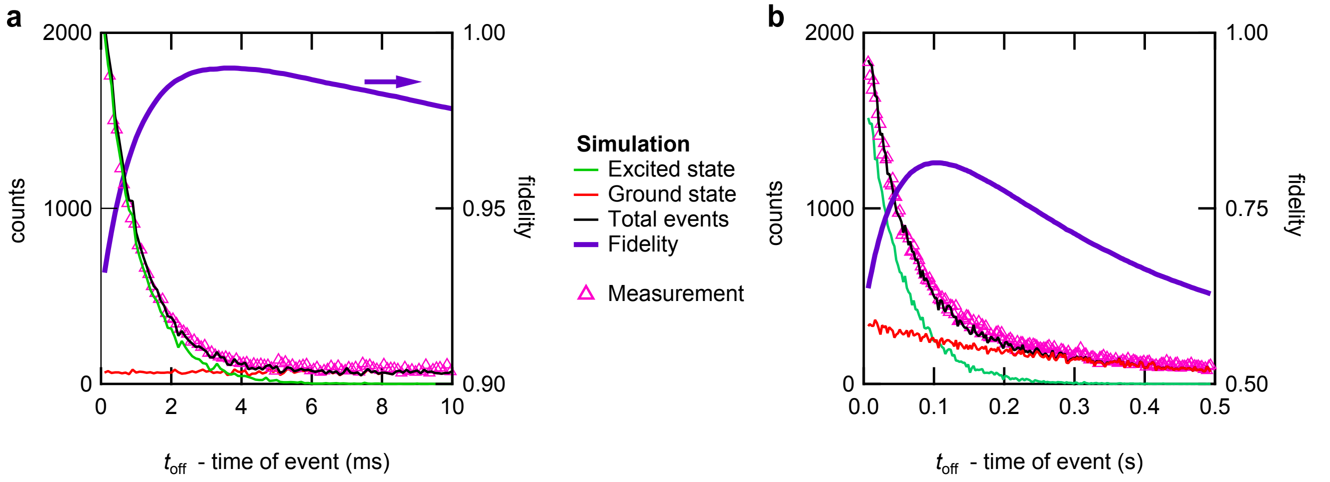

Supplementary Figure 6. T_1 Spin-readout fidelity. Simulated distribution of tunneling events in the read out stage for electrons tunneling out of the spin excited (green) and spin ground state (red) using the parameters extracted from the experiment for a dataset of **a** 4 T and **b** 0.7T. The simulated total count (black curve) agrees well with the experimental data (purple triangles). The fidelity (blue curve) is calculated by assigning all events tunneling out of the quantum dot before a certain $t_{\rm off}$ as excited state events and all other as spin ground state events and extracting the number of positive classified events from the simulated data set.

Supplementary Note 5. DEFINITIONS AND NOTATIONS FOR THE ELECTRON, NUCLEAR SPINS, AND PHONONS

We describe the quantum dot and the spin relaxation by the following model. The total electronic Hamiltonian is

$$H = T + V + H_Z + H_{\rm SOI} + H_{\rm HF}, \qquad \text{(Supplementary Equation 11)}$$

the components of which we now discuss. To this end, we define the coordinate system along the crystallographic directions by defining unit vectors $\mathbf{x} \equiv [100]$, $\mathbf{y} \equiv [010]$, and $\mathbf{z} \equiv [001]$ and the corresponding coordinates x, y, and z. The heterostructure is grown along $\mathbf{z}$, and the wavefunction corresponding to the lowest 2DEG subband is $\psi_0(z)$. In the xy-plane, the electronic states are defined by the kinetic energy with the electron effective mass m, the anisotropy tensor $\mathcal{M}$, and a bi-quadratic confinement potential,

$$T + V = \frac{1}{2m}\mathbf{p}\cdot\mathcal{M}\cdot\mathbf{p} + \frac{\hbar^2}{2m}\left(\frac{(\mathbf{r}\cdot\mathbf{n_1})^2}{l_1^4} + \frac{(\mathbf{r}\cdot\mathbf{n_2})^2}{l_2^4}\right). \qquad \text{(Supplementary Equation 12)}$$

The tensor $\mathcal{M}$ reflects the orbital effects of strong in-plane magnetic fields. It is diagonal in coordinate system with the first axis along the in-plane component of the magnetic field and the second perpendicular to it. In these coordinates $\mathcal{M}^{-1} = \mathrm{diag}(1, 1 + \Phi^2)$, so that the mass along the in-plane field is unchanged, and perpendicular to it is enhanced. The enhancement grows with Φ, the flux penetrating the 2DEG due to the field (see below). The confinement soft and hard potential axes, $\mathbf{n_1}$ and $\mathbf{n_2}$, respectively, are slightly rotated, by angle $\delta \approx 6^\circ$, with respect to the device axes [110], and [$\bar{1}$10]. The confinement lengths l_1 and l_2 are related to the excitation energies by

$$E_1 = \hbar^2/ml_1^2, \qquad \text{(Supplementary Equation 13)}$$

and an analogous equation for index 2.

The electron is subject to spin-dependent interactions. These comprise, first, the Zeeman term,

$$H_Z = \mu_F \boldsymbol{\sigma}\cdot\mathbf{B}, \qquad \text{(Supplementary Equation 14)}$$

where $(\sigma_x, \sigma_y, \sigma_z) = \boldsymbol{\sigma}$ is the vector of sigma matrices, $\mathbf{B} = B(\cos\xi\cos\phi, \cos\xi\sin\phi, \sin\xi)$ is the magnetic field, and $\mu_F = (g/2)\mu_B$ is the reduced electron magnetic moment, with the g-factor g, and the Bohr magneton μ_B. The associated Zeeman energy is $\epsilon_z = g\mu_B B = 2\mu_F B$. Second, the spin-orbit interactions. We split them to the linear Rashba and Dresselhaus terms,

$$H^{(1)}_{\rm SOI} = \alpha\left(\sigma_y p_x - \sigma_x p_y\right) + \beta\left(-\sigma_x p_x + \sigma_y p_y\right), \qquad \text{(Supplementary Equation 15)}$$

and the cubic Dresselhaus term,

$$H^{(3)}_{\rm SOI} = \frac{\gamma_c}{\hbar^3}\left(\sigma_x p_x p_y^2 - \sigma_y p_y p_x^2\right). \qquad \text{(Supplementary Equation 16)}$$

The linear interactions' strengths are parameterized by spin-orbit length $l_{\rm so}$, and angle ϑ by writing $\alpha = (\hbar/2ml_{\rm so})\cos\vartheta$, and $\beta = (\hbar/2ml_{\rm so})\sin\vartheta$. The linear spin-orbit terms can be recast, by a unitary transformation of the Hamiltonian, into the effective interaction[11],

$$H^{\rm eff}_{\rm SOI} = \mu_F(\mathbf{n}_{\rm so}\times\mathbf{B})\cdot\boldsymbol{\sigma}, \qquad \text{(Supplementary Equation 17)}$$

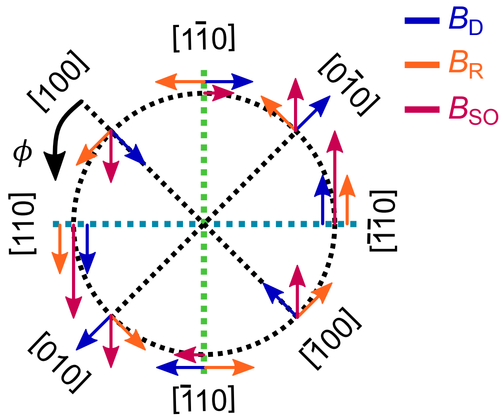

Supplementary Figure 7. Angular spin-orbit interaction. The total SOI field $B_{\mathrm{SOI}} = B_R + B_D$ (red) along various crystal axes from the interplay of the Rashba B_R (orange) and linear Dresselhaus B_D (dark blue) SOI components. The interplay of B_R and B_D leads to an anisotropic B_{SO}. A maximal $B_{\mathrm{SO}} \sim |\alpha + \beta|$ is expected along [110] and minimal $B_{\mathrm{SO}} \sim |\alpha - \beta|$ along [1$\bar{1}$0]. ϕ is defined as the angle with respect to [100].

which will be convenient below. The dimensionless spin-orbit vector

$$\mathbf{n}_{\mathrm{so}}(\mathbf{r}) = \frac{x}{l_{\mathrm{so}}}[\sin\vartheta, -\cos\vartheta, 0] + \frac{y}{l_{\mathrm{so}}}[\cos\vartheta, -\sin\vartheta, 0]. \quad \text{(Supplementary Equation 18)}$$

We write it using the dot coordinates as

$$\mathbf{n}_{\mathrm{so}}(\mathbf{r}) = \mathbf{n}_{\mathrm{so}}^{(\mathbf{1})}(\mathbf{n_1}\cdot\mathbf{r}) + \mathbf{n}_{\mathrm{so}}^{(\mathbf{2})}(\mathbf{n_2}\cdot\mathbf{r}), \quad \text{(Supplementary Equation 19)}$$

by defining the following vectors

$$\mathbf{n}_{\mathrm{so}}^{(\mathbf{1})} = \frac{1}{l_{\mathrm{so}}}[\sin(\delta+\vartheta), -\cos(\delta-\vartheta), 0], \quad \text{(Supplementary Equation 20a)}$$

$$\mathbf{n}_{\mathrm{so}}^{(\mathbf{2})} = \frac{1}{l_{\mathrm{so}}}[\cos(\delta+\vartheta), \sin(\delta-\vartheta), 0]. \quad \text{(Supplementary Equation 20b)}$$

For later convenience the following expressions are noted,

$$|\mathbf{n}_{\mathrm{so}}^{(\mathbf{1})} \times \mu_F \mathbf{B}|^2 = \left(\frac{\mu_F B}{l_{\mathrm{so}}}\right)^2 \left\{\cos^2\xi \cdot [\cos\phi\cos(\delta-\vartheta) + \sin\phi\sin(\delta+\vartheta)]^2 + \sin^2\xi\cdot(1+\sin 2\delta\sin 2\vartheta)\right\},$$

$$|\mathbf{n}_{\mathrm{so}}^{(\mathbf{2})} \times \mu_F \mathbf{B}|^2 = \left(\frac{\mu_F B}{l_{\mathrm{so}}}\right)^2 \left\{\cos^2\xi \cdot [\cos\phi\sin(\delta-\vartheta) - \sin\phi\cos(\delta+\vartheta)]^2 + \sin^2\xi\cdot(1-\sin 2\delta\sin 2\vartheta)\right\}.$$

(Supplementary Equation 21)

These expressions are anisotropic, due to the anisotropy of the spin-orbit interactions, (Supplementary Equation 15), illustrated in Supplementary Figure 7.

Third, there is Fermi's contact interaction,

$$H_{\mathrm{HF}} = A v_0 \sum_n \delta(z-z_n)\delta(\mathbf{r}-\mathbf{r}_n)\boldsymbol{\sigma}\cdot\mathbf{I}_n. \quad \text{(Supplementary Equation 22)}$$

Here, n labels the nuclei with spin $\mathbf{I}_n$ and position $\mathbf{R}_n \equiv (\mathbf{r}_n, z_n)$, and similarly $\mathbf{R} = (\mathbf{r}, z)$ is the three dimensional electron position operator. Further, A is a material constant, and $v_0 = a_0^3/8$ is the volume per atom, with a_0 the

lattice constant. To evaluate the matrix elements $H_{\rm HF}$, one has to consider also the extension of the electronic state along the $\mathbf{z}$ axis. We define the length scale l_h by[12]

$$l_h^{-1} = \int \mathrm{d}z\, |\psi_0(z)|^4, \qquad \text{(Supplementary Equation 23)}$$

which therefore depends on the 2DEG width along the growth direction. The flux due to the in-plane field is also related to the 2DEG width, by

$$\Phi = \frac{e}{\hbar}\lambda_z^2 B \cos\xi, \qquad \text{(Supplementary Equation 24)}$$

through another effective length λ_z. Both l_h and λ_z are of the order of the nominal width of the 2DEG, l_z, with the precise relation dependent on the heterostructure confinement profile. For $\psi_0(z)$, we use the ground state of a triangular potential (the Airy function), as described in detail in Ref. 13.

The electron-phonon interaction is described by

$$H_{\rm ph} = \sum_{\lambda\boldsymbol{\kappa}} \left(b_{\lambda\boldsymbol{\kappa}} + b^\dagger_{\lambda-\boldsymbol{\kappa}} \right) H_{\rm ph}^{\lambda\boldsymbol{\kappa}}, \qquad \text{(Supplementary Equation 25)}$$

where $\lambda \in \{l, t_1, t_2\}$ is the acoustic phonon branch index, with l the the longitudinal and t_1, t_2 the two transversal branches of acoustic phonons, $\boldsymbol{\kappa}$ is the three dimensional phonon wavevector, and the coupling

$$H_{\rm ph}^{\lambda\boldsymbol{\kappa}} = \sum_\eta \sqrt{\frac{\hbar\kappa}{2\rho V c_\lambda}} \sigma_\eta M^\eta_{\lambda\boldsymbol{\kappa}} e^{i\boldsymbol{\kappa}\cdot\mathbf{R}}. \qquad \text{(Supplementary Equation 26)}$$

For later notational convenience the index $\eta \in \{\rm df, pz\}$ labels here the electron-phonon interactions, deformation and piezoelectric. Further, ρ is the material density, V is the crystal volume, c_λ is the sound velocity, $M^{\rm df}_{\lambda\kappa} = \delta_{\lambda l}$ with the latter being the Kronecker delta symbol, $\sigma_{\rm df} = \sigma_e$ is the deformation potential, $\sigma_{\rm pz} = -ieh_{14}/\kappa$, with h_{14} being the piezoelectric constant, and

$$M^{\rm pz}_{\lambda\kappa} = \frac{2}{\kappa^2}\left(\kappa_x\kappa_y e^z_\lambda + \kappa_z\kappa_x e^y_\lambda + \kappa_y\kappa_z e^x_\lambda \right), \qquad \text{(Supplementary Equation 27)}$$

is a dimensionless factor defined by the components of $\mathbf{e}_\lambda$, the three mutually perpendicular polarization vectors of unit length.

Supplementary Note 6. THE SPIN RELAXATION RATE

The relaxation rate between an initial electronic state i and the final state f, with the corresponding energies E_i, E_f, corresponding to a single phonon emission, is given by the Fermi's Golden rule

$$\Gamma = \frac{2\pi}{\hbar}\sum_{\lambda\kappa} |\langle f|H_{\rm ph}^{\lambda\kappa}|i\rangle|^2 \delta\left(E_{if} - \hbar c_\lambda \kappa\right)\left[n(E_{if})+1\right], \qquad \text{(Supplementary Equation 28)}$$

where we assumed $E_{if} = E_i - E_f > 0$, and n is the phonon thermal occupation factor

$$n(\epsilon) = \frac{1}{\exp(\epsilon/k_{\rm B}T) - 1}, \qquad \text{(Supplementary Equation 29)}$$

with $k_{\rm B}$ the Boltzmann constant and T the temperature.

For the spin relaxation, the initial state is $|i\rangle = |\Psi_{0\downarrow}\rangle$, the orbital ground state with spin down, the final state is $|f\rangle = |\Psi_{0\uparrow}\rangle$, the orbital ground state with spin up, and the transition energy equals to the Zeeman energy, $E_{if} = \epsilon_z$. In the continuum limit for phonons, $\sum_{\boldsymbol{\kappa}} \to [V/(2\pi)^3]\int {\rm d}\boldsymbol{\kappa}$, we get (Supplementary Equation 28) in the following form

$$\Gamma = [n(\epsilon_z)+1]\sum_{\eta\lambda}\int {\rm d}\boldsymbol{\kappa}\frac{\kappa}{8\pi^2\rho c_\lambda}|\sigma_\eta|^2|M^\eta_{\lambda\boldsymbol{\kappa}}|^2|\tau(\boldsymbol{\kappa})|^2\delta\left(\epsilon_z - \hbar c_\lambda\kappa\right), \qquad \text{(Supplementary Equation 30)}$$

where we introduced

$$\tau(\boldsymbol{\kappa}) = \langle\Psi_{0\downarrow}|e^{i\boldsymbol{\kappa}\cdot\mathbf{R}}|\Psi_{0\uparrow}\rangle, \qquad \text{(Supplementary Equation 31)}$$

as the matrix element of the electron-phonon interaction between the initial and final state. Even though our numerics implements the evaluation of these formulas exactly, to substantiate the discussion in the main text introduction, we also provide analytical results. To this end, we adopt some approximations, most importantly the dipole approximation, expanding the exponential in (Supplementary Equation 31) to the lowest order. The quantity $|\tau|^2$ is then given by the dipole matrix element between the lowest spin opposite quantum dot states and is bilinear in the components of vector $\boldsymbol{\kappa}$ (see Supplementary Note 8). To proceed with such an expression, we define the following average

$$\langle f(\boldsymbol{\kappa})\rangle = \int {\rm d}\boldsymbol{\kappa}|M^\eta_{\lambda\boldsymbol{\kappa}}|^2 f(\boldsymbol{\kappa})\delta\left(\epsilon_z - \hbar c_\lambda\kappa\right), \qquad \text{(Supplementary Equation 32)}$$

as the integral over phonon wavevectors with the weights from (Supplementary Equation 30). The zinc-blend crystal symmetry gives the following result

$$\langle(\boldsymbol{\kappa}\cdot\mathbf{n})(\boldsymbol{\kappa}\cdot\mathbf{m})\rangle = C^\eta_\lambda\frac{\kappa_\lambda^4}{\hbar c_\lambda}(\mathbf{n}\cdot\mathbf{m}), \qquad \text{(Supplementary Equation 33)}$$

for $\mathbf{n}$ and $\mathbf{m}$ being in-plane unit vectors, $\kappa_\lambda = \epsilon_z/\hbar c_\lambda$, and the numerical constants $C_l^{\rm df} = 2\pi/3$, $C_l^{\rm pz} = 8\pi/35$, and $C_{t_1}^{\rm pz} = C_{t_2}^{\rm pz} = 2/3\times C_l^{\rm pz}$. We now write the rate as

$$\Gamma = \gamma\langle d^2\rangle, \qquad \text{(Supplementary Equation 34)}$$

splitting it to the phonon part and the (averaged) dipole moment between the spin opposite states. The first is

$$\gamma = [n(\epsilon_z)+1]\sum_{\eta\lambda}C^\eta_\lambda\frac{\kappa_\lambda^5}{8\pi^2\hbar\rho c_\lambda^2}|\sigma_\eta|^2 = \frac{n(\epsilon_z)+1}{15\pi\hbar\rho}\left(\frac{5}{4}\frac{\sigma_e^2}{\hbar^5 c_l^7}\epsilon_z^5 + \frac{(eh_{14})^2}{\hbar^3\bar{c}^5}\epsilon_z^3\right), \qquad \text{(Supplementary Equation 35)}$$

where we defined a weighted phonon velocity

$$\frac{1}{\overline{c}^5} = \left(\frac{3}{7} \frac{1}{c_l^5} + \frac{4}{7} \frac{1}{c_t^5} \right). \qquad \text{(Supplementary Equation 36)}$$

The second, derived in Supplementary Note 8, is

$$|d_{\rm SOI}|^2 \approx \quad |\mu_F \mathbf{B} \times \mathbf{n}_{\rm so}^{(\mathbf{1})}|^2 l_1^4 \frac{E_1^2}{(E_1^2 - \epsilon_z^2)^2} + |\mu_F \mathbf{B} \times \mathbf{n}_{\rm so}^{(\mathbf{2})}|^2 l_2^4 \frac{E_2^2}{(E_2^2 - \epsilon_z^2)^2}, \qquad \text{(Supplementary Equation 37a)}$$

$$|d_{\rm HF}|^2 \approx \quad \frac{2I(I+1)}{3} \frac{A^2}{N} \left(l_1^2 \frac{E_1^2}{(E_1^2 - \epsilon_z^2)^2} + l_2^2 \frac{E_2^2}{(E_2^2 - \epsilon_z^2)^2} \right). \qquad \text{(Supplementary Equation 37b)}$$

Equations (Supplementary Equation 35) and (Supplementary Equation 37) make the power dependence on the magnetic field explicit for any combination of the phonon interaction, with $\gamma^{\rm df} \propto B^5$ and $\gamma^{\rm pz} \propto B^3$ and the spin-dependent electron interaction, with $|d_{\rm SOI}|^2 \propto B^2$, and $|d_{\rm HF}|^2 \propto B^0$ (up to the small magnetic field orbital effects; see below). The expressions for the relaxation rates given in the Methods, Eq. (1) and (2), can be obtained by restricting to the dominant piezoelectric phonons in (Supplementary Equation 35), neglecting the Zeeman term with respect to the orbital energies, and using (Supplementary Equation 13), and for the spin-orbit interaction case also (Supplementary Equation 21).

Supplementary Note 7. ANISOTROPY OF THE HYPERFINE RELAXATION RATE

The strong anisotropy of the relaxation induced by the spin-orbit interactions played a major role in our experiment and allowed to distinguish it from the hyperfine effects. Indeed, compared to the explicitly anisotropic (Supplementary Equation 37a), the expression in (Supplementary Equation 37b) stays unchanged, as long as the dot shape is fixed. Neglecting the orbital effects of the magnetic field, this is indeed the case. In this section we estimate the small anisotropy induced by going beyond this approximation (of a purely in-plane field, and a 2DEG with zero width). We first consider the orbital effects of a purely in-plane field, and then an out-of-plane field. For both of these it is useful to consider a change of the mass in the kinetic term of a linear harmonic oscillator, $m \to m^*$. With the Hamiltonian written in the form of (Supplementary Equation 12),

$$\frac{p^2}{2m} + \frac{\hbar^2}{2m}\frac{r^2}{l^4} \quad \to \quad \frac{p^2}{2m^*} + \frac{\hbar^2}{2m}\frac{r^2}{l^4} \equiv \frac{p^2}{2m^*} + \frac{\hbar^2}{2m^*}\frac{r^2}{l^{*4}}, \qquad \text{(Supplementary Equation 38)}$$

Upon such a change the oscillator energy and dipole elements rescale to

$$E^* = E\left(\frac{m}{m^*}\right)^{1/2}, \text{ and } \quad l^* = l\left(\frac{m}{m^*}\right)^{1/4}. \qquad \text{(Supplementary Equation 39)}$$

the latter following from $E^* = \hbar/m^* l^{*2}$, the standard relation for the LHO energy. The in-plane field orbital effects will lead to such changes along the axis perpendicular to the magnetic field. If the dot is anisotropic, this will lead to anisotropic change of the dipole moment $|d_{\mathrm{HF}}|^2$. We quantify the magnitude of such anisotropy by the ratio of the difference and sum, of the dipole moment extrema (as a function of the magnetic field direction), which are achieved with the magnetic field along the potential axes,

$$\Delta^{\mathrm{in}}_{|d|^2} \equiv \frac{|d(\mathbf{B} \parallel \mathbf{n_1})|^2 - |d(\mathbf{B} \parallel \mathbf{n_2})|^2}{|d(\mathbf{B} \parallel \mathbf{n_1})|^2 + |d(\mathbf{B} \parallel \mathbf{n_2})|^2} = \frac{1-(1+\Phi^2)^{3/2}}{1+(1+\Phi^2)^{3/2}}\frac{E_1^{-3} - E_2^{-3}}{E_1^{-3} + E_2^{-3}}. \qquad \text{(Supplementary Equation 40)}$$

The left hand equality sign is a definition, and the right hand side was obtained by neglecting the Zeeman energy in (Supplementary Equation 37b), and using that that the in-plane field renormalizes the mass according to $m^*/m = 1 + \Phi^2$, with the flux given in (Supplementary Equation 24). The expression in (Supplementary Equation 40) is plotted for our parameters in Supplementary Figure 8.

We now turn to the case of a magnetic field with an out-of-plane magnetic component, $B \sin\xi \neq 0$. We will consider an isotropic quantum dot, for simplicity, and define the anisotropy of the rate by comparing its value for a purely in-plane field, and a value for a finite out-of-plane component. With these two values, we define Δ for this case analogously to (Supplementary Equation 40). The orbital effects of an out-of-plane field are described by a renormalization of the confinement length, and splitting the excited states energies according to their orbital moment L. These two effects are for the two excited lowest states, $L = \pm 1$, given by

$$l^{*-4} = l^{-4} + \left(\frac{eB\sin\xi}{2\hbar}\right)^2, \text{ and } \quad E^* = \frac{\hbar^2}{ml^{*2}} \pm \frac{\hbar e}{2m} B \sin\xi. \qquad \text{(Supplementary Equation 41)}$$

Calculating Δ becomes a straightforward algebra, using the previous equation, and (Supplementary Equation 37b), and we plot the result in Supplementary Figure 8. We note that for the case of a slightly asymmetric dot, the energy effect is quenched as long as $(\hbar e/2m)B_z \ll |E_1 - E_2|$, which is the case in our experiment. Keeping only the orbital squeezing effect (the renormalization of the confinement length), we then get

$$\Delta^{\mathrm{out}}_{|d|^2} \equiv \frac{|d(\xi)|^2 - |d(\xi=0)|^2}{|d(\xi)|^2 + |d(\xi=0)|^2} \approx 3\frac{e^2B^2}{8\hbar^2}\sin^2\xi. \qquad \text{(Supplementary Equation 42)}$$

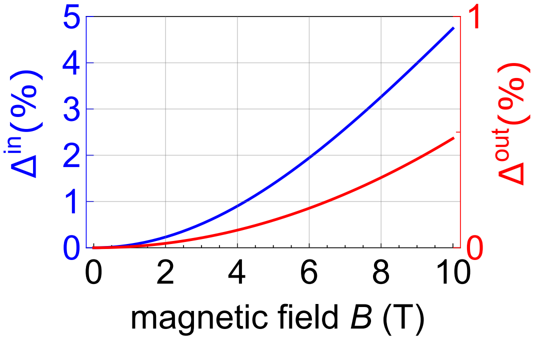

Supplementary Figure 8. Anisotropy of the hyperfine induced relaxation rate. The blue curve shows $\Delta^{\rm in}$, (Supplementary Equation 40), the in-plane anisotropy of the relaxation rate. It equals the ratio of the maximal deviation of the rate from its average, and the average, upon varying the magnetic field within the 2DEG plane. The red curve shows $\Delta^{\rm out}$, (Supplementary Equation 42), the out-of-plane anisotropy of the rate (the full expression and its approximation discussed in the text are indistinguishable on the figure resolution). It shows, again on relative scale, the change of the rate upon misaligning the field out of the 2DEG plane. We adopted the parameters of the dot, and for the second quantity we set $\xi = 1.3^\circ$, the maximal misalignment angle found in Supplementary Figure 1b.

Looking at the figure, we conclude that the expected anisotropies of the hyperfine relaxation rates due to the orbital effects of the magnetic field are indeed very small, and the hyperfine induced relaxation is therefore expected to be isotropic within the experimental resolution.

Supplementary Note 8. DIPOLE MATRIX ELEMENTS BETWEEN THE SPIN OPPOSITE STATES

We now derive (Supplementary Equation 37), considering the spin-dependent effects (other than the Zeeman energy) in the Hamiltonian (Supplementary Equation 11) perturbatively. To this end, we define the unperturbed Hamiltonian $H_0 = T + V + H_Z$ and consider the effects of the remaining terms, $H' = H_{\rm SOI} + H_{\rm HF}$, in the basis of the unperturbed eigenstates, denoted $|\Phi\rangle$, while the exact eigenstates are denoted by $|\Psi\rangle$. Both are labelled by the orbital and spin index of the electronic wavefunction, $j = 0, 1, \ldots$, and $\sigma =\uparrow,\downarrow$, respectively, and the many-particle state of the nuclear spins in the quantum dot, denoted as a multi-index μ.

With this notation, we calculate the matrix element in (Supplementary Equation 31) in the lowest order perturbation in H'. Expanding the indexes by the one corresponding to the nuclear spins, the initial state is

$$|\Psi_{0\overline{\sigma}\mu}\rangle \approx |\Phi_{0\overline{\sigma}\mu}\rangle + \sum_{j\sigma'\mu'\neq 0\overline{\sigma}\mu} \frac{\langle \Phi_{j\sigma'\mu'}|H'|\Phi_{0\overline{\sigma}\mu}\rangle}{E_{0\overline{\sigma}\mu} - E_{j\sigma'\mu'}} |\Phi_{j\sigma'\mu'}\rangle, \qquad \text{(Supplementary Equation 43)}$$

where the phonon emission (absorption) corresponds to $\sigma =\uparrow (\downarrow)$, while the final state is

$$|\Psi_{0\sigma\mu^*}\rangle \approx |\Phi_{0\sigma\mu^*}\rangle + \sum_{j\sigma'\mu'\neq 0\sigma\mu^*} \frac{\langle \Phi_{j\sigma'\mu'}|H'|\Phi_{0\sigma\mu^*}\rangle}{E_{0\sigma\mu^*} - E_{j\sigma'\mu'}} |\Phi_{j\sigma'\mu'}\rangle. \qquad \text{(Supplementary Equation 44)}$$

We assume that the unperturbed basis can be factorized

$$|\Phi_{j\sigma\mu}\rangle = |\Phi_j\rangle \otimes |\sigma\rangle \otimes |\mu\rangle, \qquad \text{(Supplementary Equation 45)}$$

so that the orbital part does not depend on the spin indexes, and that the electron-phonon interaction, the matrix element of which we are calculating, is diagonal in both spin indexes. This gives

$$\tau(\boldsymbol{\kappa}) = \sum_j \langle \overline{\sigma}\mu| \frac{e^{i\boldsymbol{\kappa}\cdot\mathbf{R}_{0j}} H'_{j0}}{E_{0j} + (E_{\sigma\overline{\sigma}} + E_{\mu^*\mu})} + \frac{e^{i\boldsymbol{\kappa}\cdot\mathbf{R}_{j0}} H'_{0j}}{E_{0j} - (E_{\sigma\overline{\sigma}} + E_{\mu^*\mu})} |\sigma\mu^*\rangle, \qquad \text{(Supplementary Equation 46)}$$

where we have introduced the notation for orbital matrix elements as

$$O_{ij} = \langle \Phi_i|O|\Phi_j\rangle, \qquad \text{(Supplementary Equation 47)}$$

for energies as $E_{ij} = E_i - E_j$, and similarly for the spin indexes. Note also that the $j = 0$ term cancels exactly from the sum in (Supplementary Equation 46).

We now adopt the dipole approximation, by expanding the electron-phonon interaction to the lowest order

$$e^{i\boldsymbol{\kappa}\cdot\mathbf{R}} \approx 1 + i\boldsymbol{\kappa}\cdot\mathbf{R}, \qquad \text{(Supplementary Equation 48)}$$

which leads to

$$\tau(\boldsymbol{\kappa}) = i\boldsymbol{\kappa}\cdot \sum_{j\neq 0} \langle \overline{\sigma}\mu| \frac{\mathbf{R}_{0j} H'_{j0}}{E_{0j} + (E_{\sigma\overline{\sigma}} + E_{\mu^*\mu})} + \frac{\mathbf{R}_{j0} H'_{0j}}{E_{0j} - (E_{\sigma\overline{\sigma}} + E_{\mu^*\mu})} |\sigma\mu^*\rangle, \qquad \text{(Supplementary Equation 49)}$$

This leads to substantial simplification for a bi-harmonic confinement. Indeed, in such case, only the lowest two excited states have non-zero dipole matrix element with the ground state, which are mutually orthogonal (even if they are complex, which is, however, not considered here). In this case, the integration over the phonon wavevectors $\boldsymbol{\kappa}$ makes the mixed terms in $|\tau|^2$ zero, see (Supplementary Equation 33). We therefore get

$$\langle |\tau(\boldsymbol{\kappa})|^2\rangle = \langle |\boldsymbol{\kappa}\cdot\mathbf{d_1}|^2\rangle + \langle |\boldsymbol{\kappa}\cdot\mathbf{d_2}|^2\rangle, \qquad \text{(Supplementary Equation 50)}$$

where the dipole moments for the excited states are given by

$$\mathbf{d_j} = \langle \overline{\sigma} \mu | \frac{\mathbf{R}_{0j} H'_{j0}}{E_{0j} + (E_{\sigma\overline{\sigma}} + E_{\mu^*\mu})} + \frac{\mathbf{R}_{j0} H'_{0j}}{E_{0j} - (E_{\sigma\overline{\sigma}} + E_{\mu^*\mu})} |\sigma\mu^*\rangle. \quad \text{(Supplementary Equation 51)}$$

Next we evaluate these dipole elements separately for the spin-orbit, and hyperfine interactions. We will also neglect the nuclear Zeeman energies $E_{\mu^*\mu}$ as negligible compared to the electron Zeeman energy $E_{\sigma\overline{\sigma}} = \overline{\sigma}\epsilon_z$, and notice that we can put $\mathbf{R}_{ij} = \mathbf{r}_{ij}$, if all considered states are from the lowest 2DEG subband, what is the case here.

Let us take first the spin-orbit interactions. We take into account only the linear terms in their effective form, $H' = H_{\mathrm{SOI}}^{\mathrm{eff}}$, and neglect the cubic term, and ignore nuclear effects, by putting $\mu^* = \mu$. Since the effective spin-orbit interaction is also of the dipole operator form, we easily get

$$|\mathbf{d_j}|^2_{\mathrm{SOI}} = |\mathbf{r}_{0j}|^4 |\mathbf{n}_{\mathrm{so}}^{(\mathbf{j})} \times \mu_F \mathbf{B}|^2 \frac{4E_{0j}^2}{(E_{0j}^2 - \epsilon_z^2)^2}. \quad \text{(Supplementary Equation 52)}$$

Using here the results for the Fock-Darwin eigenfunctions, $\mathbf{r}_{01} = (l_1/\sqrt{2})\mathbf{n_1}$, and $\mathbf{r}_{02} = (l_2/\sqrt{2})\mathbf{n_2}$, and Eq. (Supplementary Equation 50) gives Eq. (Supplementary Equation 37a).

The calculation for the hyperfine interaction proceeds analogously, and we get

$$|\mathbf{d_j}|^2_{\mathrm{HF}} = (Av_0)^2 \sum_{mn} \left(\frac{\mathbf{r}_{0j}\delta^n_{j0}}{E_{0j} + E_{\sigma\overline{\sigma}}} + \frac{\mathbf{r}_{j0}\delta^n_{0j}}{E_{0j} - E_{\sigma\overline{\sigma}}} \right) \cdot \left(\frac{\mathbf{r}_{j0}\delta^m_{0j}}{E_{0j} + E_{\sigma\overline{\sigma}}} + \frac{\mathbf{r}_{0j}\delta^m_{j0}}{E_{0j} - E_{\sigma\overline{\sigma}}} \right) \langle \overline{\sigma}\mu | \mathbf{I}_n \cdot \boldsymbol{\sigma} | \sigma\mu^* \rangle \langle \sigma\mu^* | \mathbf{I}_m \cdot \boldsymbol{\sigma} | \overline{\sigma}\mu \rangle, \quad \text{(Supplementary Equation 53)}$$

where we denoted $\delta^n_{ij} = [\delta(\mathbf{R} - \mathbf{R}_n)]_{ij}$. The expression in (Supplementary Equation 53) depends on the initial and final state of the nuclear subsystem. The experimentally relevant situation is that these two states are not restricted in any way, which corresponds to a rate being summed over all possible final states and averaged, with the proper statistical weights, over the possible initial states,

$$\overline{|\mathbf{d}|^2} = \sum_{\mu\nu} p(\mu) |\mathbf{d}(\mu, \nu)|^2. \quad \text{(Supplementary Equation 54)}$$

A straightforward calculation for an unpolarized nuclear ensemble, $p(\mu) = \text{const}$, gives

$$\overline{\langle \sigma\mu | \mathbf{I}_m \cdot \boldsymbol{\sigma} | \overline{\sigma}\nu \rangle \langle \overline{\sigma}\nu | \mathbf{I}_n \cdot \boldsymbol{\sigma} | \sigma\mu \rangle} = \frac{2}{3} I(I+1) \delta_{nm}, \quad \text{(Supplementary Equation 55)}$$

with δ the Kronecker delta. Using this in (Supplementary Equation 53) we get

$$\overline{|\mathbf{d_j}|^2}_{\mathrm{HF}} = A^2 \frac{2}{3} I(I+1) |\mathbf{r}_{0j}|^2 \frac{4E_{0j}^2}{(E_{0j}^2 - \epsilon_z^2)^2} v_0^2 \sum_m |\Phi_0(\mathbf{R}_m)|^2 |\Phi_j(\mathbf{R}_m)|^2. \quad \text{(Supplementary Equation 56)}$$

As the linear density of the nuclear spins, $2/a_0$, is very high compared to the lengthscales of the electronic wavefunctions, l_1, l_2, l_z, the sum over nuclei can be well approximated by an integral, $v_0 \sum_m \to \int \mathrm{d}\mathbf{R}$. Defining inverse volumes as the following wavefunction overlaps

$$V_{0j}^{-1} = \int \mathrm{d}\mathbf{R} |\Phi_0(\mathbf{R})|^2 |\Phi_j(\mathbf{R})|^2, \quad \text{(Supplementary Equation 57)}$$

the harmonic model gives $V_{00} = 2\pi l_1 l_2 l_h$, and $V_{01} = V_{02} = 4\pi l_1 l_2 l_h$. Putting $N = V_{00}/v_0$ as the number of the nuclei "within" the quantum dot volume, leads to (Supplementary Equation 37b) by using (Supplementary Equation 56) in (Supplementary Equation 50).

Supplementary Note 9. NUMERICAL IMPLEMENTATION

The spin relaxation rates are obtained inserting the numerically exact eigenstates into (Supplementary Equation 28) and performing the integration over the phonon momenta numerically, by standard methods[14]. Whenever the Hamiltonian includes the hyperfine interaction, the given relaxation rate is a geometric average of rates for 1000 configurations of static nuclear spins with random orientations (the approximation of unpolarized nuclei at infinite temperature). Specifically, the rate obtained at run i is written as $\Gamma_i = \exp(\gamma_i)$, and the average rate is defined as $\Gamma_{\rm mean} \equiv \exp(\overline{\gamma})$, while the "error bar" given on such a value is defined by the maximal and minimal rates being $\Gamma_{\rm max/min} = \exp(\overline{\gamma} \pm \delta\gamma)$, with $\delta\gamma^2$ the dispersion of the exponents γ_i. This definition is chosen for convenience of resulting in a symmetric "error" interval on a logarithmic plot, so that the minimal rate stays non-zero, irrespective of the degree of the fluctuations among the individual rates. It should be taken only as a way to compare the degree of fluctuations among two values from numerics, rather than an assessment of fluctuations possibly observed in the experiment, since the latter depend in a non-trivial way on the relation between the measurement total time and the nuclear ensemble ergodic time[15].

Supplementary Note 10. PARAMETERS AND FITTING OF THE SPIN-ORBIT CONSTANTS

In the evaluation of the rates according to the above described model, we use the parameters of GaAs, $\rho = 5300\,\mathrm{kg\,m^{-3}}$, $c_l = 4784\,\mathrm{m\,s^{-1}}$, $c_\mathrm{t} = 3350\,\mathrm{m\,s^{-1}}$, $\sigma_\mathrm{e} = 7\,\mathrm{eV}$, $h_{14} = 1.4 \times 10^9\,\mathrm{V\,m^{-1}}$, $m = 0.067\,m_\mathrm{e}$, $\gamma_\mathrm{c} = 11\,\mathrm{eV\AA^3}$, $I = 3/2$, $A = 45\,\mu\mathrm{eV\,nm}$, $a_0 = 5.65\,\mathrm{\AA}$. We also estimate the electron temperature $T = 60\,\mathrm{mK}$, though the corresponding thermal factor in (Supplementary Equation 29) is negligible even for highest magnetic fields, so that the temperature plays little role for the value of the spin relaxation (it can be set to zero in (Supplementary Equation 28) leading to no visible changes). In addition to these parameters, we extract the excitation energies $E_x = 2.3\,\mathrm{meV}$ and $E_y = 2.6\,\mathrm{meV}$, corresponding to $l_x \approx 22\,\mathrm{nm}$, and $l_y \approx 21\,\mathrm{nm}$, and the g-factor $g = -0.36$, from the spectral data such as those shown on Fig. 1c–d of the main text, and their spin-resolved analogues. As noted in the above and in the main text, to match the experimental relaxation rates quantitatively, one needs further details on the dot shape. As explained in detail in Ref. 13, we fit $l_z = 6.5\,\mathrm{nm}$, and $\delta = 5.6 \pm 1^\circ$, which gives $l_h/l_z = 2.4$, $\lambda_z/l_z = 1.009$ upon assuming a triangular confinement potential along the heterostructure growth direction.

With all these values fixed, we fit the linear spin-orbit lengths by minimizing the following chi-square sum

$$\chi^2 = \sum_i \left(\ln \Gamma^{(i)}_\mathrm{theory} - \ln \Gamma^{(i)}_\mathrm{exp}\right)^2 w_i, \qquad \text{(Supplementary Equation 58)}$$

with respect to the fitting parameters l_so and ϑ. In the sum the index i runs through the whole measured dataset of the relaxation rates $\Gamma = 1/T_1$ and we take the logarithm of the rate as it spans a range of many orders of magnitude. The weights are chosen as $w_i = \ln(1.05 + \delta\Gamma^{(i)}_\mathrm{exp}/\Gamma^{(i)}_\mathrm{exp})$, with $\delta\Gamma$ the error estimated when fitting the value of Γ, as explained in Supplementary Figure 5, and 1.05 is an arbitrarily chosen factor. However, we find that the extracted values of l_so and ϑ are rather robust to many other choices (including ignoring the errors altogether). We find that the minimization converges into the following two local minima

$$l_\mathrm{so} = 2.1(1)\,\mu\mathrm{m},\ \vartheta = 31(1)^\circ, \qquad \text{(Supplementary Equation 59a)}$$

$$l_\mathrm{so} = 2.1(1)\,\mu\mathrm{m},\ \vartheta = 61(1)^\circ. \qquad \text{(Supplementary Equation 59b)}$$

where the values in the brackets give the typical error on the last given digit. These errors are estimated from the spread of the converged values upon running the minimization algorithm many times. The reason that we are not able to quantify these errors more precisely, is due to several uncertainities pertaining to the experimental as well as numerical inputs to the chi square sum. For example, the numerical value $\Gamma^{(i)}_\mathrm{theory}$ is a random variable, due to the randomness in the nuclear configuration. For the minimization, which is very computationally demanding, we are able to perform an average over typically only tens of random nuclear configurations for each i, which makes these statistical fluctuations quite large. For the same reasons, we are not able to quantify the likelihood ratio for the two local minima given in (Supplementary Equation 59). However, using again multiple runs, we conclude that the difference between the two possibilities is, within our model, statistically significant, and the value $\vartheta = 31^\circ$ fits the measured data better. Figure Supplementary Figure 9 illustrates the amount of data used to calculate χ^2 in the described minimization procedure.

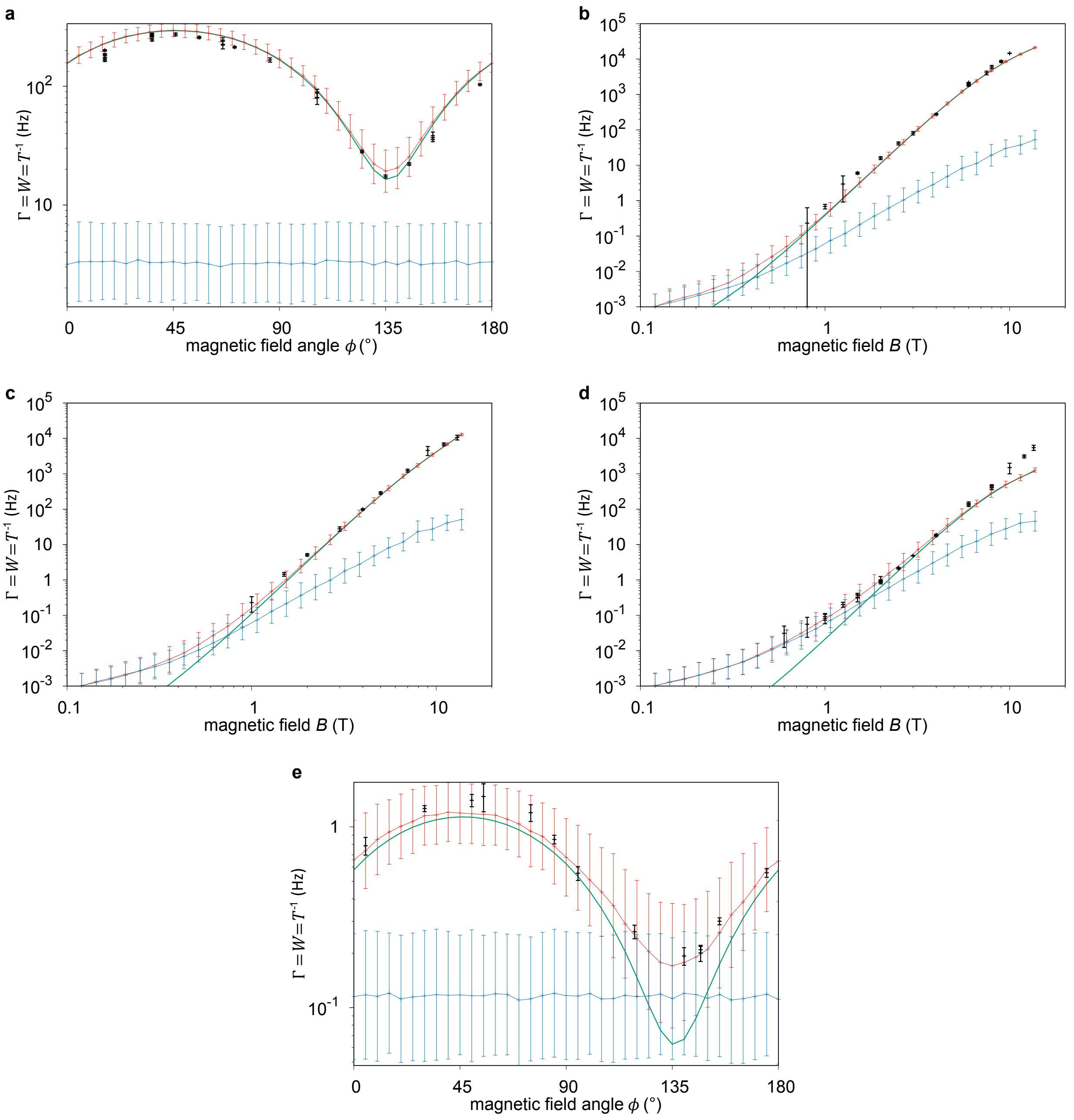

Supplementary Figure 9. The total set of T_1 data used to fit the spin-orbit parameters. In all panels, we show the measured spin relaxation rates (black points with error bars) versus the theoretical values (lines) for the full model (red), the model excluding the hyperfine interactions (green) and the model excluding the spin-orbit effects (blue). The plotted values were obtained for $\vartheta = 31.3°$ and $l_{\rm so} = 2.08$ μm and illustrate a single step in the minimization routine. Typically less than hundred steps are needed for convergence. In panel **a**–**e**, the following parameters are fixed: **a** $B = 4$ T, **b** $\phi = 45°$, **c** $\phi = 356°$, **d** $\phi = 315°$, **e** $B = 1.25$ T. Error bars in the calculated data is from geometric average of rates for 1000 configurations of static nuclear spins with random orientations (see Supplementary Note 9). Error bars on experimental data are fitting errors.

Supplementary References

---

[*] These authors contributed equally to this work

[1] Amasha, S. *et al.* Measurements of the spin relaxation rate at low magnetic fields in a quantum dot Preprint at `http://arxiv.org/abs/cond-mat/0607110` (2006).

[2] Nazarov, Y. V. & Blanter, Y. M. *Quantum Transport* (Cambridge University Press, Cambridge, 2009).

[3] Gustavsson, S. *et al.* Counting Statistics of Single Electron Transport in a Quantum Dot. *Phys. Rev. Lett.* **96**, 076605 (2006). URL `https://link.aps.org/doi/10.1103/PhysRevLett.96.076605`.

[4] MacLean, K. *et al.* Energy-Dependent Tunneling in a Quantum Dot. *Phys. Rev. Lett.* **98**, 036802 (2007). URL `https://link.aps.org/doi/10.1103/PhysRevLett.98.036802`.

[5] Amasha, S. *et al.* Spin-dependent tunneling of single electrons into an empty quantum dot. *Phys. Rev. B* **78**, 041306 (2008). URL `https://link.aps.org/doi/10.1103/PhysRevB.78.041306`.

[6] Stano, P. & Jacquod, P. Spin-dependent tunneling into an empty lateral quantum dot. *Phys. Rev. B* **82**, 125309 (2010). URL `http://link.aps.org/doi/10.1103/PhysRevB.82.125309`.

[7] House, M. G. *et al.* Detection and Measurement of Spin-Dependent Dynamics in Random Telegraph Signals. *Phys. Rev. Lett.* **111**, 126803 (2013). URL `https://link.aps.org/doi/10.1103/PhysRevLett.111.126803`.

[8] Yamagishi, M., Watase, N., Hashisaka, M., Muraki, K. & Fujisawa, T. Spin-dependent tunneling rates for electrostatically defined GaAs quantum dots. *Phys. Rev. B* **90**, 035306 (2014). URL `https://link.aps.org/doi/10.1103/PhysRevB.90.035306`.

[9] Morello, A. *et al.* Single-shot readout of an electron spin in silicon. *Nature* **467**, 687– 691 (2010). URL `http://www.nature.com/articles/nature09392`.

[10] Elzerman, J. M. *et al.* Single-shot read-out of an individual electron spin in a quantum dot. *Nature* **430**, 431–435 (2004). URL `http://www.nature.com/doifinder/10.1038/nature02693`.

[11] Baruffa, F., Stano, P. & Fabian, J. Theory of anisotropic exchange in laterally coupled quantum dots. *Phys. Rev. Lett.* **104**, 126401 (2010). URL `https://link.aps.org/doi/10.1103/PhysRevLett.104.126401`.

[12] Merkulov, I. A., Efros, A. L. & Rosen, M. Electron spin relaxation by nuclei in semiconductor quantum dots. *Phys. Rev. B* **65**, 205309 (2002). URL `http://link.aps.org/doi/10.1103/PhysRevB.65.205309`.

[13] Stano, P. *et al.* Gate-defined quantum dot in a strong in-plane magnetic field: orbital effects. Preprint at `https://arxiv.org/abs/1804.00128` (2018).

[14] Press, W. H., Teukolsky, S. A., Vetterling, W. T. & Flannery, B. P. *Numerical Recipes 3rd Edition: The Art of Scientific Computing* (Cambridge University Press, New York, NY, USA, 2007), 3 edn.

[15] Delbecq, M. R. *et al.* Quantum Dephasing in a Gated GaAs Triple Quantum Dot due to Nonergodic Noise. *Phys. Rev. Lett.* **116**, 046802 (2016). URL `https://link.aps.org/doi/10.1103/PhysRevLett.116.046802`.
